# Posterior Vertebral Pedicular Tethering for the Treatment of Idiopathic Adolescent Scoliosis

**DOI:** 10.3390/healthcare11131878

**Published:** 2023-06-28

**Authors:** Jorge Mineiro

**Affiliations:** Orthopaedic Spine Unit, Department of Orthopaedics and Traumatology, Hospital CUF Descobertas, 1998-018 Lisbon, Portugal; jorge.mineiro@cuf.pt

**Keywords:** posterior vertebral pedicular tethering, idiopathic adolescent thoracolumbar and lumbar scoliosis

## Abstract

Over the last decade, there has been a new wave of interest in non-fusion techniques for the treatment of adolescent idiopathic scoliosis. These are not new techniques, as they were first published and presented in the late 1950s, using compression of the convexity or distraction of the concavity of the main curvature. More recently, anterior vertebral body tethering has raised great interest, as although it is a major procedure through the child’s chest, it seems appropriate for the thoracic curves. The main objective of this article is to describe Posterior Vertebral Pedicular Tethering (PVPT) as a “new” technique performed as a less invasive spinal procedure for the treatment of certain thoracolumbar and lumbar scoliosis in growing adolescents. It is an alternative growth modulation technique appropriate for thoracolumbar and lumbar curvatures where we observe reduction of the three plane deformity of idiopathic scoliosis in adolescents.

## 1. Introduction

Idiopathic scoliosis in adolescents (AIS) is a spinal three-dimensional deformity that presents as lateral coronal deviation, rotation of the vertebral bodies towards the concavity, and reduced thoracic kyphosis. This type of scoliosis has the tendency to progress with growth, and if the Cobb angle reaches 45° or higher, it is often taken as an indication for surgery, as these curvatures continue to aggravate even after skeletal maturity [1] and may lead to a reduction in lung volume [2]. Three-dimensional correction of curvature in the different planes and allowing continued spinal growth has been the main objective of the treatment of scoliosis in a child who is still growing [3]. Over the last fifty years, posterior segmented instrumented fusion with pedicle screws has been the traditional method to address these abnormal spinal curvatures [4], although it is not yet the ideal procedure for a growing young adolescent.

Although clinical outcome studies over the years have shown encouraging results in terms of spinal deformity correction and long-term patient satisfaction, the end result of spinal fusion surgery is a permanent, irreversible, absolute reduction in spinal mobility. This change in motion induces alterations on the mechanical loading of adjacent mobile segments [5,6], which may cause problems at a later stage in adult life with disc degeneration [7,8].

These findings have motivated surgeons over the last century to investigate and search for alternative treatment options that might preserve spinal motion and growth and maintain intervertebral disc height and properties while correcting the scoliotic deformity. The goal will be to provide a means for these adolescents to harness their remaining spinal growth to produce a correction of the deformity in the different planes.

These are procedures commonly known as “growth friendly,” where the implants allow the spine to grow either by distracting the concavity of the main curve at regular intervals, by allowing the growing spine to slide along the implants, or by compressing the convexity of the curvature during growth of the spine.

### 1.1. Historical Background

Spinal tethering is not new. Over the last century, several experimental procedures have been attempted, although they are not clearly inspired by biomechanics or by thorough knowledge of the complex phase of growth spurt in adolescence. I am sure that several attempts have been made by orthopaedic surgeons who in the 1950s, 1960s, and 1970s were dealing with adolescent idiopathic scoliosis. One of them was Adam Gruca from Poland, who in 1956 published in France his innovative techniques for treating AIS in the *Revue de Chirurgie Orthopedique et Traumatologique* [9], and, in 1958, presented them in the USA and published in *JBJS-A* [10].

**Figure 1:**
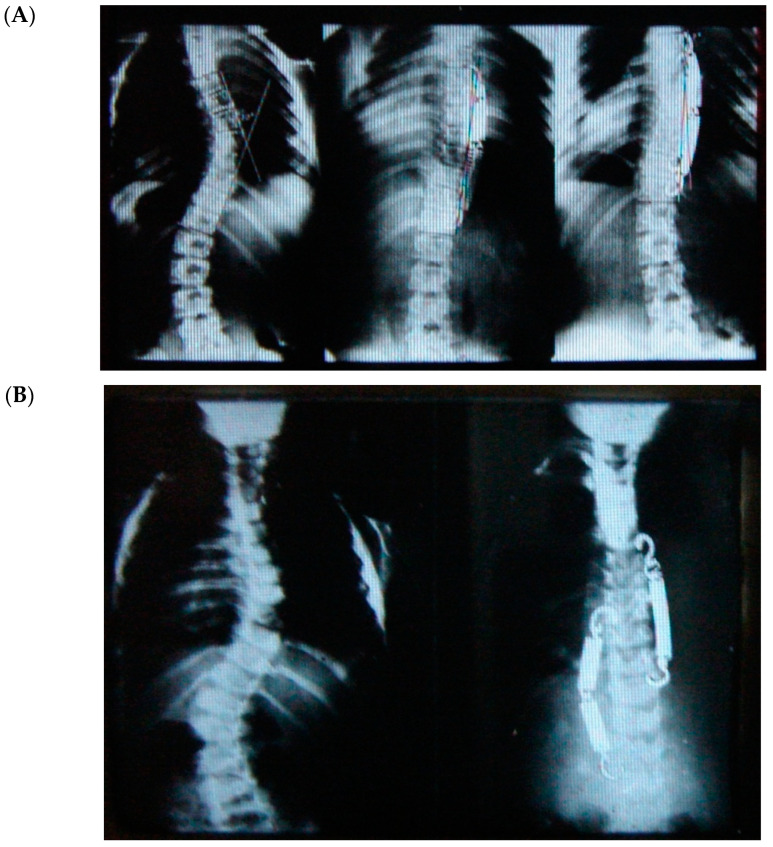
(**A**) Thoracic scoliosis treated with Gruca Springs. (**B**) Double curve scoliosis treated with Gruca Springs.

I was a medical student when my father died in 1978. The late Jorge Draper Mineiro was an orthopaedic surgeon, but one of that generation who dealt with pathologies covering all subspecialities, from children to adults and from spines to hips and feet deformities. Concerned about how the spine would grow and how deformities would develop in humans, he focused most of his research on “the blood supply of the vertebral column” from infancy to adulthood. That was his PhD thesis, which was published in 1965 (he was the first SRS Portuguese member). As a medical student, I remember my father speaking about this technique that a friend had taught him for the treatment of adolescent scoliosis with metal springs—it was a much less aggressive procedure, there were fewer complications and risks compared to the traditional Harrington rods they used, and they would not have to fuse the spine with this technique. He passed away in 1978, but last year, when I had to empty his study at home, I went through his archives of thousands of slides and pictures and I found the technique he had mentioned years ago for treating adolescent idiopathic scoliosis. As you can imagine, there was not much information on each case apart from the slides of the radiographs pre- and post-op at follow up (FU). This innovative technique (tethering) used by him in the early 1960s was performed from the posterior approach using the device developed by his friend, A. Gruca (Gruca Springs). These were sterile springs in metal (empty and not covered/protected) with a hook at each end to anchor on the transverse processes. As you can see from Figure 1, you could use one or more attached to each other, depending on the length of the convexity. Analyzing the few cases, it sounded like it would go from end to end vertebrae. However, these springs did break frequently, but my father commented that the spine would not lose the correction achieved. He was surprised and intrigued by the findings, which he could not explain until he revised some cases and found that inside the empty metal spring was a fibrous tether that would replace the mechanical effect of the device. Therefore, based on these findings, he operated on a series of patients using, as a tether, a very long animal tendon. Unfortunately, I have no record of these cases.

From Mineiro’s archives (Figure 1), we can see that the posterior tether did work on the coronal plane for thoracic, thoracolumbar, and even double curvature scoliosis, but at that stage, orthopaedic surgeons were not concerned about the sagittal profile. Therefore, there are no lateral radiographs from these patients. In summary, looking back into the past, we can say that the posterior tether works for correcting the coronal scoliotic deformity in the adolescent growing spine (thoracic and thoracolumbar), but what would happen to the hypokyphosis of the spine in the lateral plane?

A decade later, in the 1970s, deformity surgeons were more concerned with the correction of scoliosis in both planes: coronal and lateral (sagittal). Alan Dwyer [11] and, later, Klaus Zielke [12] introduced the treatment of adolescent scoliosis by instrumenting the spine anteriorly. However, they soon pointed out that their results in the lumbar curves were poor, and they were the first to draw attention to the fact that anterior spinal instrumentations are kyphotic and ideal for thoracic curves where you need to create kyphosis in the hypokyphotic thoracic segment. For the lumbar spine, their results were very bad, and the kyphotic effect was only overcome later by the use of wedged structural grafts/cages in the intervertebral spaces, which created lordosis and also improved the high pseudarthrosis rate they had obtained in their series [13,14,15].

Evidence from the past suggests that posterior tethering does work to correct the coronal deformity in adolescent scoliosis, and that anterior instrumentations to correct scoliosis are kyphotic, which is ideal for the thoracic spinal deformity.

### 1.2. Spinal Growth Modulation

The growth plate of vertebral bodies of the human growing spine, between C3 and L5, is located on its superior and inferior endplates. Vertebral growth is obtained by two simultaneous physiologic processes: endochondral (length) and membranous/appositional (volume) ossification [16] through the thin growth plates and from paired neurocentral, articular processes and single spinous process synchondrosis, posteriorly. Longitudinal growth of the vertebrae occurs not only anteriorly through the growth plates, but also posteriorly in each of the posterior elements [17].

As we know, distraction of the growth plate promotes, and compression inhibits, growth [18] in different regions of the axial skeleton, according to the Hueter–Volkmann principle. The use of staples or unilateral plates to induce asymmetrical growth plate inhibition in growing children with genu valgum or varum has been used for decades to correct lower extremity mechanical axial deviations [19]. However, if the staples/plates are misplaced too anteriorly or too posteriorly to the sagittal axis, it will produce an associated flexion or extension deformity, respectively.

The application of unilateral compressive forces to modulate the growth of vertebral bodies is thought to act at the level of the vertebral growth plate. Chay et al. found that experimentally tethering a pig’s spine created a scoliotic spinal deformity, resulting in a decrease in proliferative zone height of the growth plate on the side of the tether compression compared to the contralateral side of the vertebrae [20]. The cartilage cell numbers and the hypertrophic zone height within this zone also decrease following a unilateral compression of the vertebral body [21,22].

Based on these findings, surgeons have attempted asymmetrical hemiepiphysiodesis to correct abnormal spinal growth, although results have remained rather poor or unpredictable [23,24] in the treatment of spinal deformities of the growing spine.

More recently, a similar technique used staples over the disc and vertebral growth plates. However, only in minor thoracic curves less than 35° was it successful, and in these cases, scoliosis is usually treated with orthosis in growing adolescents [25]. However, a moving spine often led to mechanical complications, as it resulted in loosening of the implants extending over the intervertebral disc [26].

Spinal tethering, although not a new technique, seems to correct scoliosis three-dimensionally in certain adolescent patients without the use of spinal fusion. It uses the patient’s own spinal growth to gradually improve the curve correction achieved after the surgical procedure, thus preserving spinal mobility between the different vertebrae by applying the same Hueter–Volkmann principle.

The effects of skeletal maturity (using hand radiograph) on post-operative growth modulation was investigated by Alanay et al. [27]. They concluded that growth modulation was unpredictable in Sanders 1 reaching up to 45°, and in Sanders 2 up to 29°, of growth modulation post-operative. Sanders 3–5 were the most predictable in terms of growth modulation in their series of Vertebral Body Tethering (VBT).

The premise of growth modulation in the spine is supported by experimental studies and basic science. Asymmetric mechanical compression of vertebral body growth plates can slow the convex side growth (anterior and lateral aspect of the vertebral body) of the spinal curvature [28,29,30] while still allowing posterior synchondrosis/elements to grow. Under the same principle, asymmetrical posterior compression applied unilaterally to vertebral neurocentral synchondrosis will also inhibit growth of the convex aspect of the spinal column (posterior elements), but in this case, growth of the vertebral body anteriorly is allowed to proceed. These findings reinforce the concept of why anterior instrumentation in a growing spine will also produce kyphosis and, when applied posteriorly, why it will induce lordosis.

### 1.3. Biomechanics of Anterior Instrumentation in the Spine

Anterior spinal instrumentation causes compression of the anterior and middle column of the spine, thereby shortening the anterior vertebral column and elongating the posterior column, as described by Dennis [31]. Shortening the anterior column of the spine may help to restore kyphosis in the thoracic spine, but the literature has shown that this kyphotic effect has very poor results in the thoracolumbar and lumbar curves [32]. This is particularly true at the stage when the instrumentation in the front is a cable or a single rod that is not too rigid [11,12].

This relevant drawback of anterior spine instrumentation was only overcome with subsequent developments in the instrumentation and the devices’ design by understanding the relevance of thoracic kyphosis and lumbar lordosis in the sagittal balance of the spine and by understanding how to achieve stable correction and fusion, particularly in segments distal to the thoracic kyphosis. The introduction of new screw/rod systems with stiff rods (single and double) and bicortical vertebral body screws allowed for a new correction maneuver; correction of the deformity was achieved by rotation of a pre-bent stiff rod and by filling in the increased intervertebral space in the thoracolumbar and lumbar spine with a wedge-shaped tricortical bone graft or cages.

De-rotation of the scoliotic vertebrae through an anterior rigid fixation could be achieved by the insertion of screws into the vertebral body with different angulations that are attached to the molded rigid rod, which is subsequently de-rotated to adjust the “normal” sagittal profile of that segment of the spine.

For all these reasons, it is difficult to change the biomechanics of the spine in order to adjust a flexible anterior instrumentation of the thoracolumbar and lumbar spine to be able to create lordosis.

Biomechanics of the spine with scoliosis treated by posterior segmental instrumentation have shown us that if you want to create kyphosis, you distract between the instrumented segments, and if you want to create lordosis, you compress the segments [33]. In the growing spine, growth modulation through posterior unilateral compression of the neurocentral synchondrosis slows growth on the convex posterior side of the curvature, correcting not only scoliosis but also inducing lordosis.

### 1.4. Indication for Surgery

#### 1.4.1. Anterior Tethering

Up to the present, a single major thoracic curve (Lenke 1A or 1B curve) with non-structural lumbar or proximal thoracic curve in a preadolescent patient [26,34,35] is the most well-documented indication for spinal tethering from an anterior approach. In a recent article, Krakow et al. [36] reported on how many AIS patients would be suitable candidates for VBT. Approximately 25% of their patients fulfilled the growth parameters and curve characteristics (Lenke 1, 3, 5, or 6 curves, excluding structural upper thoracic curve) in order to be candidates for VBT. The other 75% of patients with scoliosis may still require posterior spinal instrumented fusion.

Skeletal growth in AIS children can be evaluated using the hand radiograph (Sanders’ classification) [37]. Adolescents with a right thoracic curve (40° to 60°), who are relatively flexible (30° or less on bending), who have a rib hump measuring less than 20° on the scoliometer, and who have an acceptable amount of remaining spinal growth (Sanders 3, 4, or 5) are the ideal candidates for anterior tethering procedures [26,35].

As a growth-friendly procedure, choosing the correct timing for VBT is extremely relevant. The patient with enough growth may undergo over-correction (right-sided curve turns into left-sided curve) if the procedure is carried out too early. Additionally, if performed too late, the remaining growth modulation may not correct the curve enough and/or the tether may rupture. Towards the end of adolescence, for patients with limited growth remaining or no growth at all (Sanders 6, 7, or 8), this type of procedure is not indicated.

Alanay et al. [27] pointed out in 2020 that the behavior of curve correction after VBT in adolescents with thoracic scoliosis varies according to the Sanders stages. Patients who are rated Sanders 2 were more prone to develop over-correction, and patients rated Sanders 3, 4, or 5 were less likely to develop mechanical complications. These findings reinforce the need to accurately assess Sanders growth stages and to choose the details on how much correction should be achieved at the end of the surgical tethering procedure in order to avoid complications.

#### 1.4.2. Posterior Tethering

Vertebral Pedicular Tethering (VPT) is performed from the posterior approach using a less invasive technique. This is a procedure that we started using nearly two years ago upon revisiting the results of idiopathic adolescent scoliosis treated with Gruca Springs in the 1960s and after obtaining Ethics Committee approval (Hospital CUF Descobertas Ethics Committee—Projecto/estudo 182).

The VPT procedure Is Ideal for cases with thoracolumbar and lumbar (Lenke 5C) hypolordotic scoliotic curvatures. Patients with flexible curves, a Cobb angle between 40 and 60° (correctible to 50% or less on bending), and a suitable amount of remaining growth (Sanders 3, 4, or 5) are most suitable for this technique.

The appropriate timing for VPT is also extremely important. If carried out too early or with too much correction on the table, the patient with enough growth may undergo over-correction, and if performed too late (Sanders 6 or 7) or if under-corrected on the table, the remaining growth modulation may not correct the curve enough and/or the tether may rupture.

#### 1.4.3. Anterior and Posterior Tethering

Upon patient selection, surgeons dealing with both tethering techniques, anterior or posterior, will have to match and play with the Sanders stage, the type of deformity, and the degree of curve correction on the table at the original procedure in order to avoid over- or under-correction. However, despite Alanay’s recommendations [27], there are no accurate guidelines regarding these two items on how to proceed. It is a challenge in this field of uncertainty to adjust surgical correction and to the estimate the amount of remaining growth and growth-dependent correction to obtain the expected result.

At present, spinal tethering is one of the options to treat AIS without arthrodesis of the spine, regardless of whether it is performed through an anterior or a posterior approach depending on the type of curvature. This approach has been possible with better understanding of the biomechanics of the different spinal segments, technical developments in minimally and less invasive techniques, and improved instrumentation and device design [34,37,38,39]. The tethering implants applied either to the vertebral bodies laterally [26] or to the pedicles unilaterally mechanically restrict scoliosis progression by restraining the remaining spinal growth on the convexity of the main curvature.

Although we addressed deformity correction in the coronal and sagittal planes, there is one plane of the scoliotic deformity, rotation, which also improves with growth modulation in both posterior and anterior tethering. Mechanically, it is more difficult to explain how this happens without the segmental vertebral de-rotation maneuvers (either single rod or direct vertebral body de-rotation) we use in posterior segmental spinal fusion [33], but in fact it does happen with both tethering techniques, as reported by A. Alanay et al. [27] and also in our case series.

**Figure 2:**
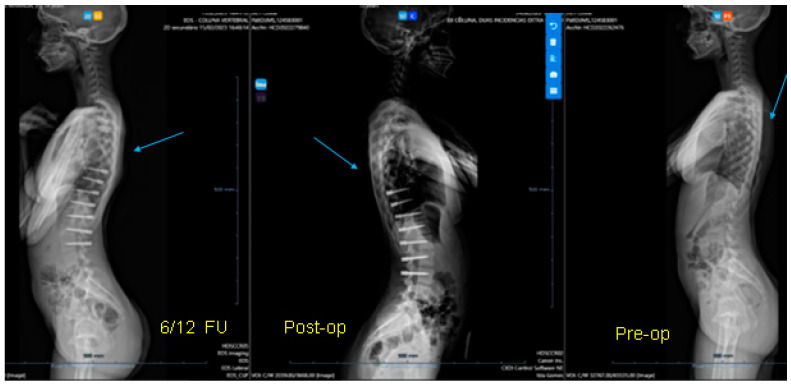
Radiological regression of rib hump with decrease of the prominent ribs.

Correction of two (scoliosis and hypokyphosis) of the three plane deformities in scoliosis has already been explained, but how rotational correction happens in these growth-friendly procedures is not clear. We know that with the progression of scoliosis, the rotation of the vertebral bodies towards the concavity increases due to the imbalance/disturbance of the different growth plates and synchondrosis in the growing spine. Tethering, either anterior or posterior, on the convexity of the curvature locks this progressive abnormal rotation and may allow the opposite side to de-rotate the vertebrae gradually with growth, thereby improving the rotational deformity and the rib hump, as shown (Figure 2).

## 2. Material and Methods

### 2.1. Demographics

Over the last 1.5 years, 6 female adolescents underwent VPT. They were all postmenarcheal, and 3 of these girls refused conservative treatment.

We present data on the 3 cases with more than 6/12 FU in order to be able to assess growth modulation (the other 3 cases had an FU of less than 6 months).

### 2.2. Radiographic and Skeletal Maturity Data

**Figure 3:**
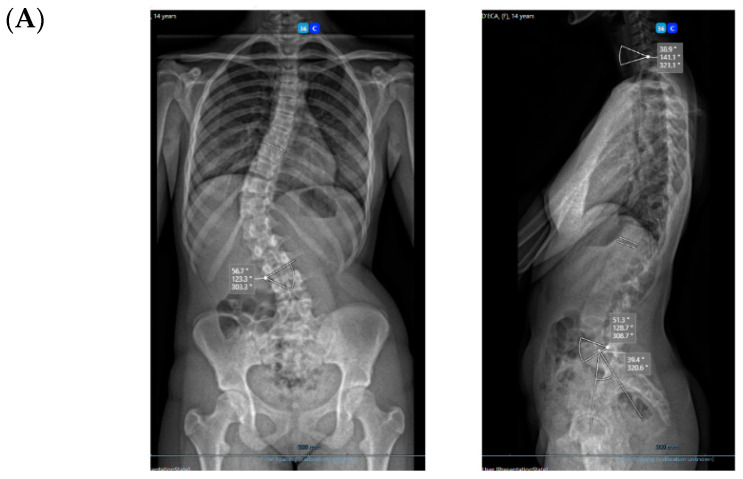

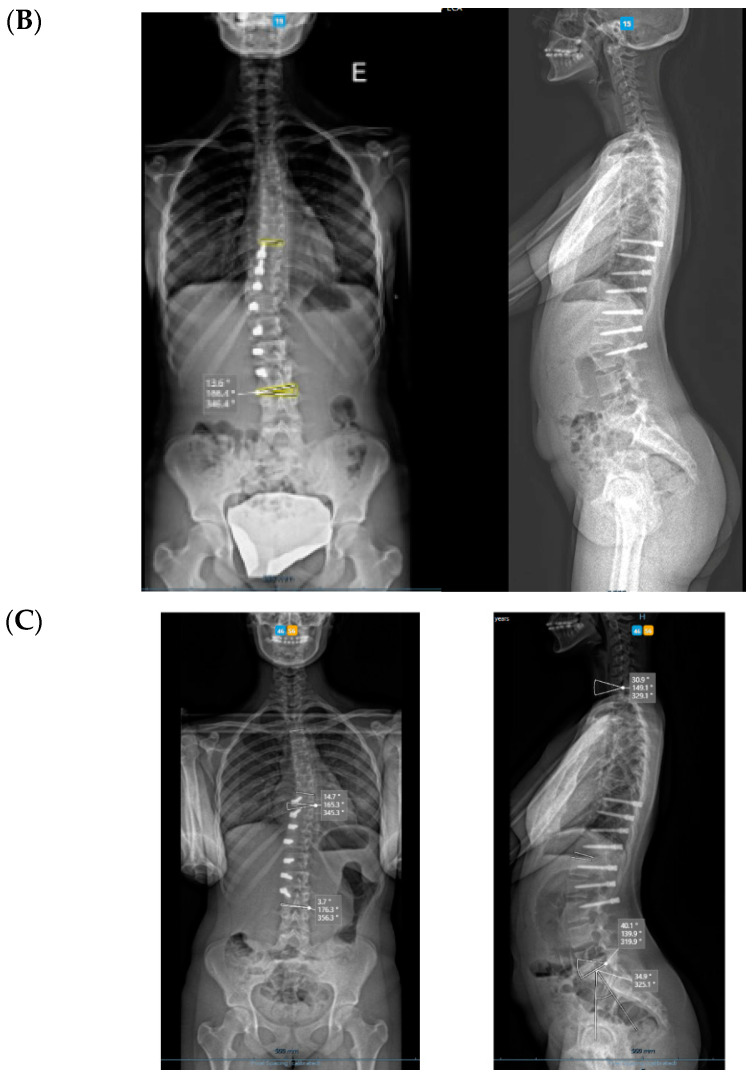
A 14-year-old adolescent. (**A**) Pre-op. (**B**) Immediate post-op. (**C**) One-year follow up.

**Figure 4:**
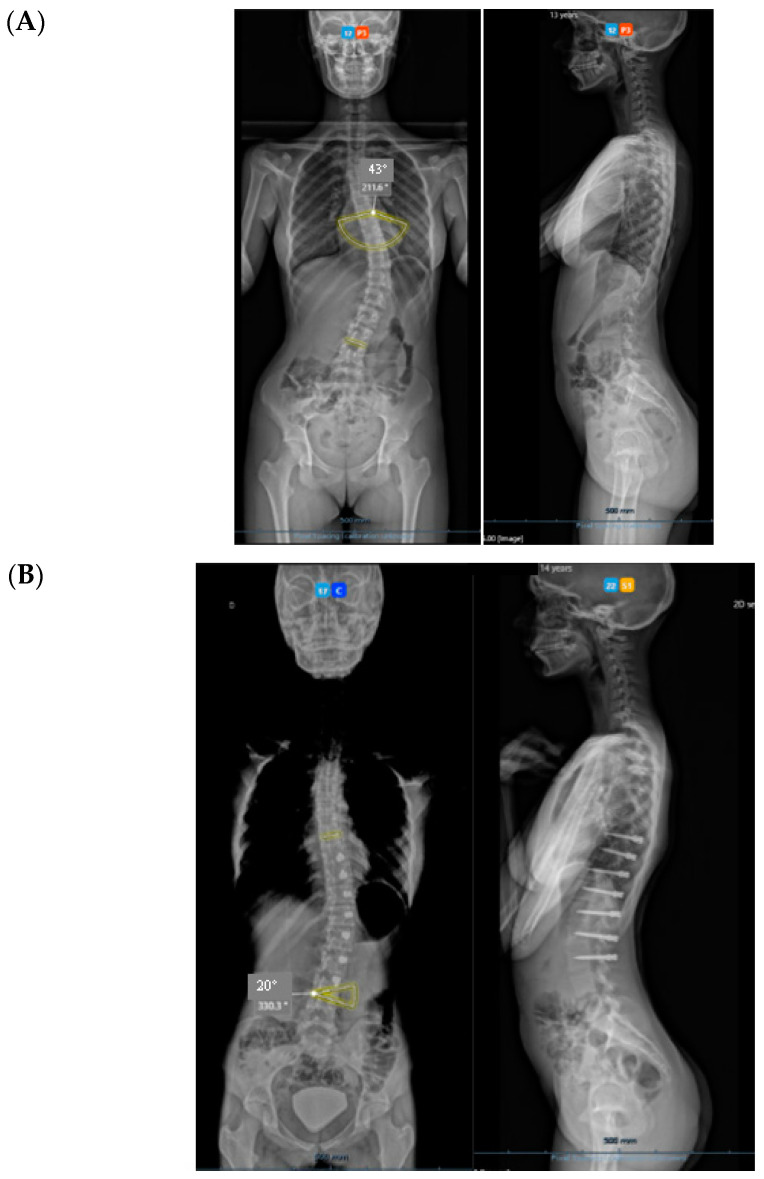
A 13-year-old adolescent. (**A**) Pre-op. (**B**) At 6-month follow up.

**Figure 5:**
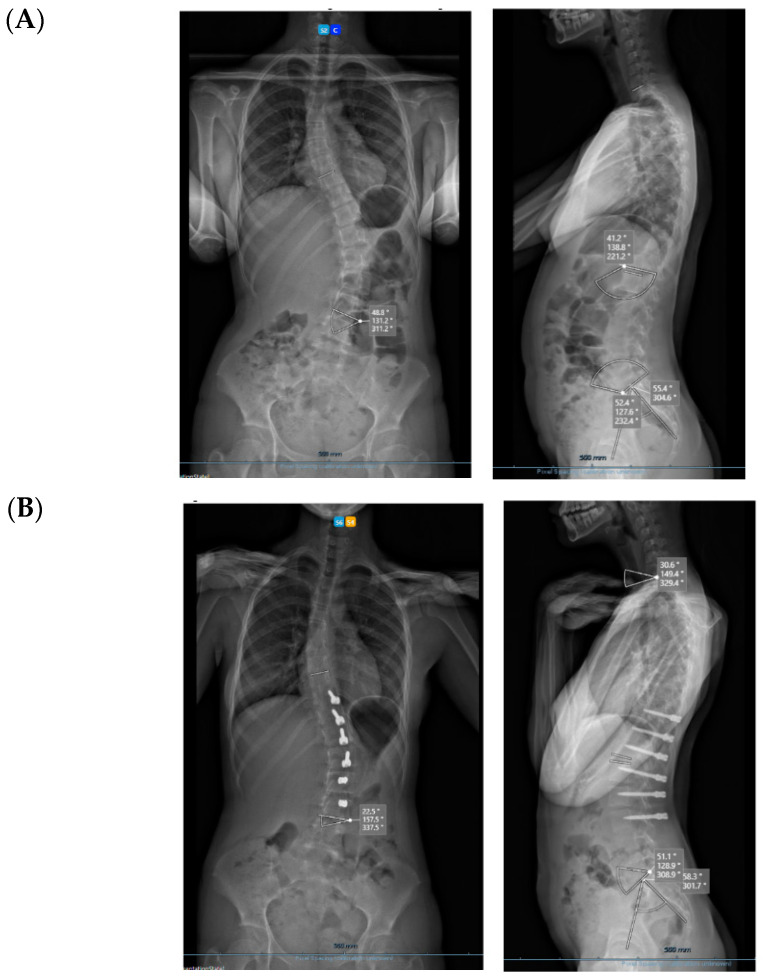
A 14-year-old adolescent. (**A**) Pre-op. (**B**) At 6-month follow up.

All three cases were thoracolumbar scoliosis (Lenke 5C) with an apex between T12 and L1 (Figure 3, Figure 4 and Figure 5).

As far as skeletal maturity is concerned, two cases were Sanders 3 (Risser 1 and 0) and one case was Sanders 5 (Risser 4).

## 3. Results

**Figure 6:**
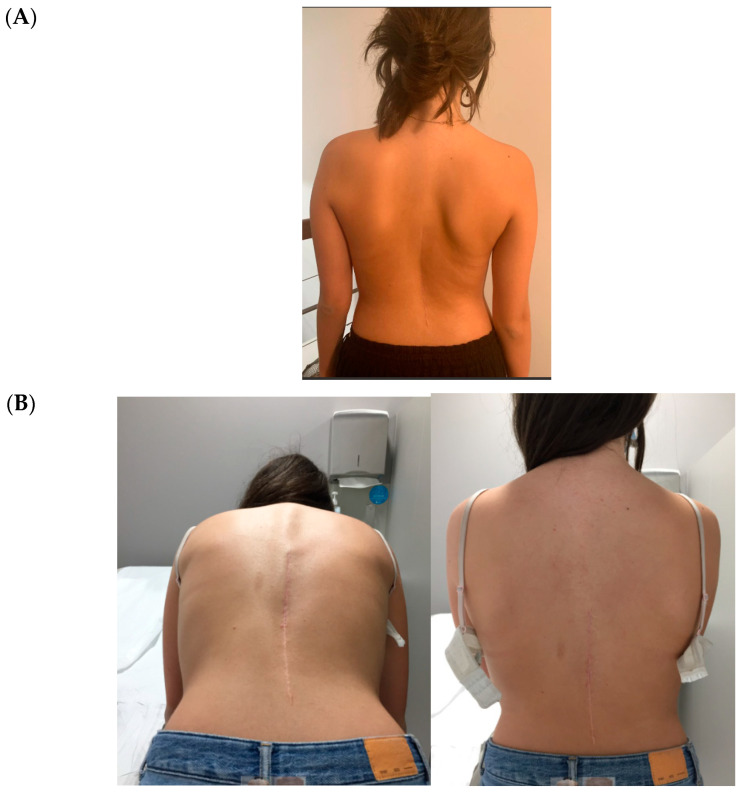
Rib hump at 6 months (**A**) and 18 months (**B**). Follow up shows progressive improvement.

All cases have progressed well with improvement of the curvatures with growth (Table 1) both in the coronal and sagittal alignment. At the 6-month FU, there was a mean 67.5% (34.8°) improvement in the main Cobb angle, but at 1 y, the oldest case over-corrected, and this was clinically noted by the girl, who pointed out that her flank symmetry had recently inverted (from one side to the other) after being symmetrical for some time (Figure 6).

Regarding the sagittal profile, we have looked at three different segments—T1–T9, T10–L3, and L3S1. In this 6-month period, the proximal segment in these three cases had increased kyphosis by 7.7°; the intermediate segment had increased lordosis by 10.9°; and the distal lumbar segment had decreased lordosis by 13.6°. However, in the only case that underwent tether release due to coronal over-correction, it is interesting that the thoracic kyphosis increased, the intermediate lordosis decreased, and the distal lumbar lordosis increased post-operatively; as such, spinal sagittal balance was well-maintained.

**Figure 7:**
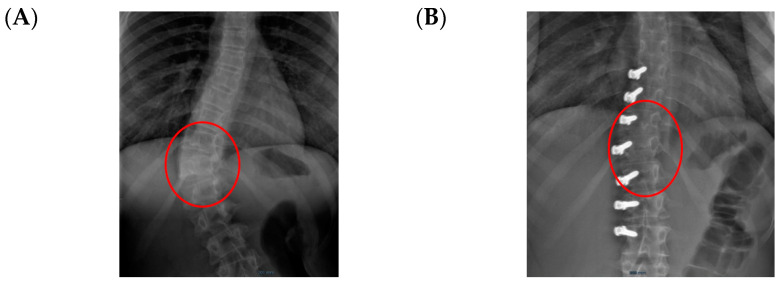
Apical improvement with progressive de-rotation of the vertebrae. (**A**) Pre-op. (**B**) FU at 1 year.

Concerning de-rotation, we can assess the apical vertebral rotation on the pre-operative radiograph as a Cobb grade +3 (Nash–Moe 50%) that improved to a Cobb grade +1 (Nash–Moe of 0%) in the last FU film (Figure 7) one year later.

As we know, vertebrae from C3–L5 have five secondary ossification centers that appear at puberty. One is at the tip of the spinous process, one is at the tip of the transverse process on each side, and the two ring or annular epiphysis is at the upper and lower surface of the vertebral bodies. By placing pedicular screws under compression on the convexity attached to the tether (not a rigid rod but a flexible screw head–tether angle), we will decrease growth at the posterolateral “corner” of the annular growth plates. This process will reverse the pathologic rotational deformity towards the concavity in main curve, while at the same time allowing the front of the spine to grow (thus improving hypolordosis). We are now pursuing an engineering modelling project to understand more accurately how this de-rotation mechanism develops.

**Figure 8:**
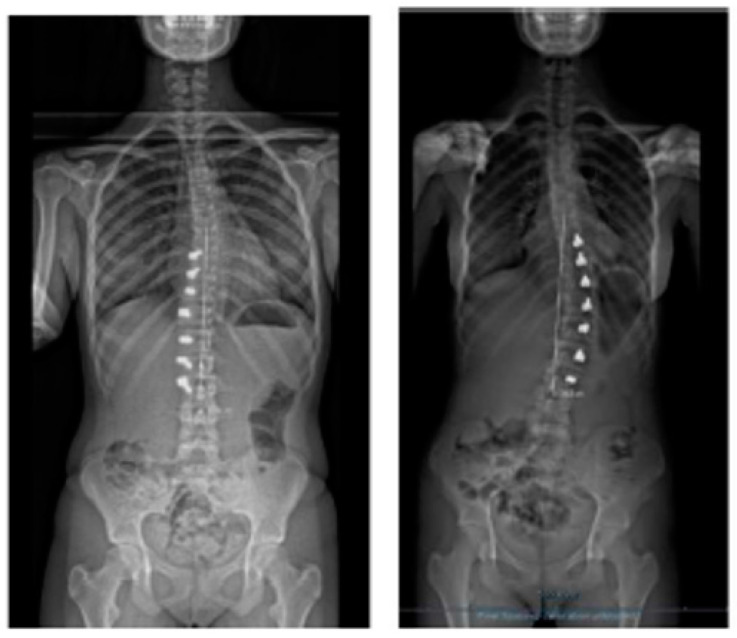
Measuring “spinal length” between two spots in two different spines shows the difficulty in interpretation.

**Figure 9:**
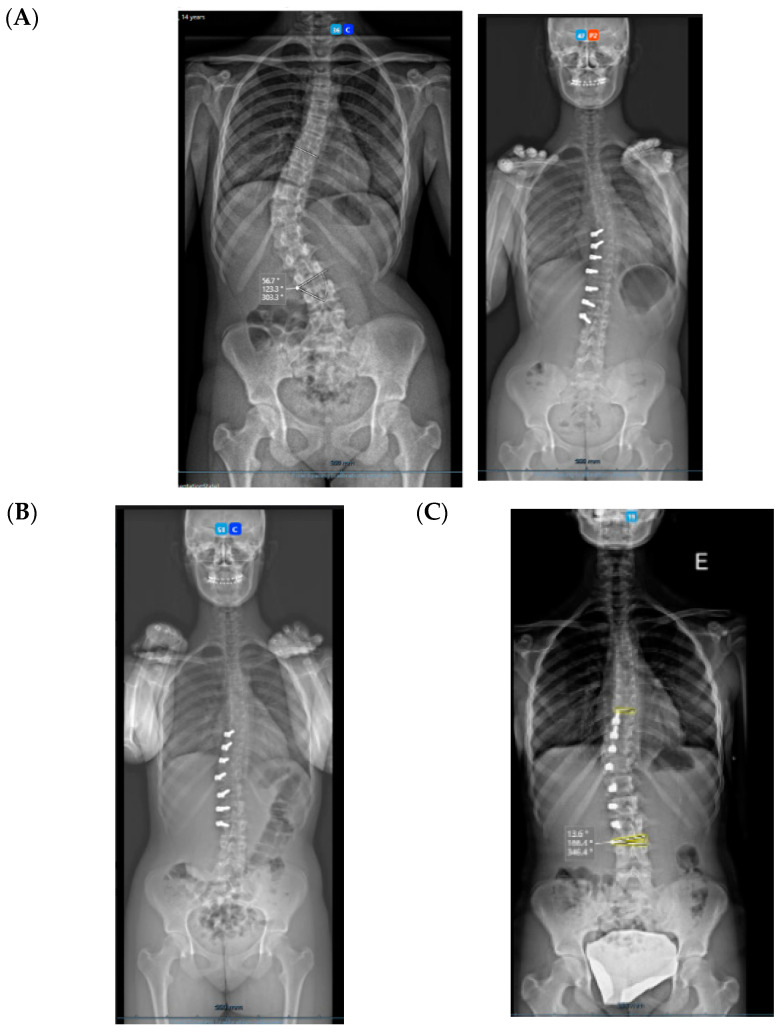
Over-correction. (**A**) Pre-op (**left**) + 18-month FU (curvature apex corrects to the opposite side) (**right**). (**B**) Post tether release. (**C**) Immediate post-op erect radiograph.

Spinal growth is difficult to assess accurately in a coronal deformity that is gradually improving by the surgical procedure in a spine that is still growing, as the two chosen spots will gradually distance away from each other through these two mechanisms. We have taken the same midpoint in the distal endplate of the instrumented vertebra or on the proximal endplate, which is always on the same proximal junctional vertebra (Figure 8). Over this period of 6 months, the referred distance has increased by a mean of 7 mm (min. 3–max. 16 mm) in the three cases, and the oldest girl (56.7° Cobb angle pre-op) saw the greatest increase. In the case of such flexible scoliosis with a more severe Cobb angle that corrected significantly with this technique (Figure 8 and Figure 9), it is obvious that the two chosen spots will distance more from each other than in scoliosis with a smaller Cobb angle. This increase in spinal length between the two endplate spots is not spinal growth per se, but differentiating these two mechanisms is difficult.

### 3.1. Complications

The only case with a 15-month follow up progressed to coronal over-correction and had to be revised (under local anaesthetic) for tether release at four levels (Figure 9A,B). Since then, she has been doing well clinically and radiologically. This complication may have happened because correction obtained at the index procedure was too much, with a Cobb angle of 13.6° in the immediate post-operative erect radiograph (pre-op Cobb angle 56.7°) in a girl rated Sanders 3 (Figure 9C).

### 3.2. Surgical Technique

In the pre-operative clinic, patients and their families were counseled that this was an “off-label” technique to treat AIS, and that the risks were essentially failure to obtain an acceptable correction at the end of growth that could require revision to a standard posterior spinal instrumented fusion.

All patients had normal pre-operative spinal MRIs and the surgical procedure was performed under spinal cord monitoring. Under the same general anaesthetic, an AP traction spinal radiograph was taken to assess the flexibility of the different curves.

**Figure 10:**
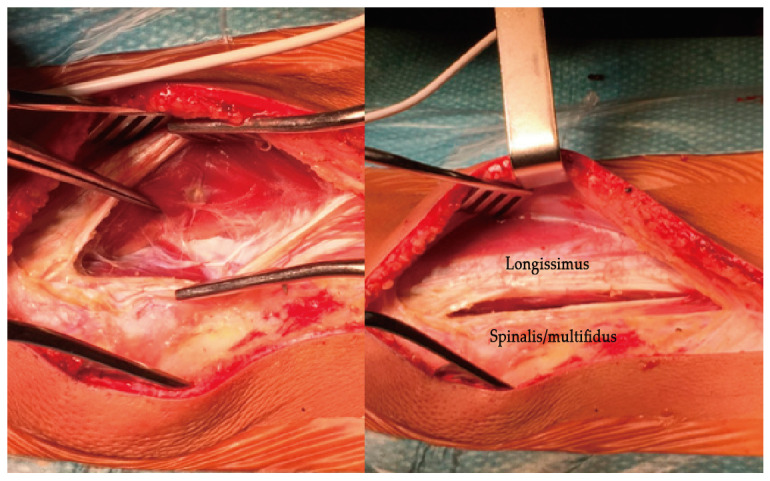
Dissection between muscles.

Under general anaesthetic and with the patient positioned prone, the spine is approached by a single posterior midline incision over the 6 or 7 segments between the end vertebrae. By using a single posterior midline incision, you avoid any extra incisions in the spine if you need to revise the instrumentation for any reason (unacceptable over-correction, under-correction, or progression of the deformity requiring standard posterior instrumented fusion). Upon opening the skin, you dissect the subcutaneous tissue approximately 1 to 2 cm from the midline towards the side of the curve convexity. You then palpate the edge of the articular processes and open vertically the fascia at the same length. Find the gap between the longissimus and the spinalis muscles (Figure 10) and palpate the spots for the insertion of the pedicular screws with minimal dissection. Insert the pedicle screws (with or without fluoroscopy) lateral to the articular processes but more or less at the same level so that it does not disturb the biomechanics of the moving facet joints.

**Figure 11:**
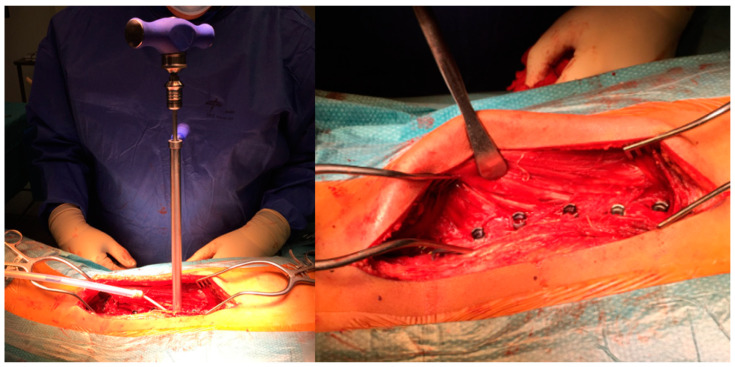
Tensioning the tether.

A polyethylene tether is inserted in the screw heads and tightened starting cranially. Tightening the tether between the screws is performed using a specific device for force measurement so that compression on the vertebrae of the convexity is applied, but not uniformly (Figure 11). According to the technique, the apical segments are tightened into 300–400 N (maximum), and then decrease the force gradually towards the proximal and the more distal screws to a maximum of 150–200 N in order to prevent screw pull-out and to correct the deformity only partially. The tension in the upper and distal segments of the instrumentation is often very slight or sometimes even left slack. Beware that just by laying the patient on the operating table under general anaesthetic, the flexible curves reduce a great deal, so it is very easy to over-correct the deformity with these powerful instruments; otherwise, you may need to help by pushing the convexity before tightening the tether. By applying translation and de-rotation on the convexity apex before tensioning the tether, you are avoiding undue stress on the screws at this early stage.

The main objective of these maneuvers is to achieve a 15° to 20° curve on the table in order to have approximately 25° to 30° on the post-operative X-ray when the patient is upright.

Intraoperative blood loss is usually minimal, averaging 40 to 80 mL. The operative time varies between 60 and 90 min, and the length of hospital stay ranges from 3 to 5 days post-operatively.

### 3.3. The Pros and Cons of Posterior Vertebral Pedicular Tethering (PVPT)

Advantages (Pros): The main advantage of PVPT is allowing correction of the AIS deformity without fusing the spine. Initial correction is achieved with implants inserted through a posterior, less invasive midline approach. Partial correction of the deformity is obtained at the operative procedure, but further improvement is achieved through the axial growth modulation by the inserted tether. Avoiding arthrodesis of the spine preserves at least some extent of the spinal range of motion, and with better functional outcomes in the long run.

In the case of complications, where revision is required, this posterior approach has numerous other advantages—in the case of coronal or sagittal over-correction, with a minor procedure under local anaesthetic in most cases, you are able to cut the tether at the different levels you intended to. In the case of unacceptable under-correction or curve progression, you are able to approach the spine through the same skin incision and perform a posterior spinal instrumented fusion with concave side instrumentation only, which has been shown to achieve good results [40]. In the case of screw pull-out or loosening or a broken tether that requires revision, everything can be performed from the posterior approach as a less invasive procedure with no majors risks or concerns.

Upon a stable, uneventful recovery, operated adolescents are able to resume normal physical activity related to sports, although I restrict certain contact sports, such as rugby or martial arts.

Disadvantages (Cons): The most common disadvantage of PVPT is related to the fact that it is only suitable for a small group of patients: those with thoracolumbar and lumbar curves with a Cobb between 40° and 60° and Sanders stages 3 to 5 of skeletal maturity.

Although the coronal deformity in this group of selected patients is corrected adequately, when we look at the spinal sagittal profile from this small series, we can see that the PVPT technique will inevitably aggravate lordosis in the intermediate thoracolumbar segment, although within normal values and with a better sagittal balance. Stagnara et al. [41] reported that the average value between T11L3 is −5° of lordosis but with a range that varied from −30° of lordosis to +15° of kyphosis in a sample of healthy young adults.

From a technical point of view, the available set of screws for tethering procedures were developed for anterior procedures only, and screw heads are rather bulky. Using them posteriorly on the convexity of the curve, they become quite prominent in some of these small, thin adolescents, which may create problems with the overriding soft tissue cover.

Having no accurate technical guidelines to adjust the amount of correction at the index procedure to the Sanders stages of skeletal maturity and type of curvature will require longer follow up, larger series, and longer prospective studies in order to clarify these issues. Larger series and prospective studies are also needed to validate this “new” technique that was used a few decades ago, which is now being applied using the same principle but with different implants.

Another theoretical concern is the long-term sustainability of the results of this growth guided technique to treat a condition for which the gold standard of treatment for comparison is a spinal fusion.

## 4. Conclusions

Spinal fusion is still the gold standard treatment for adolescent idiopathic scoliosis that progresses despite conservative treatment. However, it is not the panacea for all growing adolescents with scoliosis for whom bracing failed. In addition, fusing a segment of the spine has several functional disadvantages.

Over the years, deformity surgeons have searched for techniques that could preserve not only spinal growth but also spinal mobility while avoiding all the disadvantages of a fused solid spinal segment.

PVPT is a promising technique that uses the same Hueter–Volkmann principle that compression inhibits growth (in this case, of the convex side of the vertebral bodies while enhancing growth at the concave side) to gradually correct the deformity in the different planes while preserving spinal growth and mobility.

This less invasive spinal surgical procedure (LISS) performed from the posterior approach can also be used for revision surgery with easy access and minimal risks.

These are early results of this new technique for the treatment of adolescent idiopathic scoliosis for adolescents who have not reached skeletal maturity. However, it is a new type of growth modulation procedure that will only benefit certain young patients with thoracolumbar or lumbar curves (Lenke 5C) within a certain stage of skeletal maturity (identified as Sanders stages 3 to 5).

In order to validate this new approach for the treatment of adolescent idiopathic scoliosis, we need larger series with longer FU.

## Figures and Tables

**Table 1:** Mean values.

	Pre-Op	6-Month FU
Sagittal angle T1-T9	+26.2°	+33.9°
Sagittal angle T10-L3	−19.9°	−30.8°
Sagittal angle L3-S1	−48.7°	−35.1°
Scoliosis Cobb angle	51.6°	16.7°
Spinal Length	18.3 cm	19 cm

## Data Availability

Not applicable.

## References

[1] Weinstein L.S., Zavala D.C., Ponseti I.V. (1981). Idiopathic scoliosis: Long-term follow-up and prognosis in untreated patients. J. Bone Jt. Surg. Am..

[2] Newton O.P., Faro F.D., Gollogly S., Betz R.R., Lenke L.G., Lowe T.G. (2005). Results of preoperative pulmonary function testing of adolescents with idiopathic scoliosis. A study of six hundred and thirty-one patients. J. Bone Jt. Surg. Am..

[3] Akbarnia B.A. (2007). Management themes in early onset scoliosis. J. Bone Jt. Surg. Am..

[4] Suk I.S., Lee S.M., Chung E.R., Kim J.H., Kim S.S. (2005). Selective thoracic fusion with segmental pedicle screw fixation in the treatment of thoracic idiopathic scoliosis: More than 5-year follow-up. Spine.

[5] Berger-Roscher N., Casaroli G., Rasche V., Villa T., Galbusera F., Wilke H.-J. (2017). Influence of Complex Loading Conditions on Intervertebral Disc Failure. Spine.

[6] Ghiselli G., Wang J.C., Bhatia N.N., Hsu W.K., Dawson E.G. (2004). Adjacent segment degeneration in the lumbar spine. J. Bone Jt. Surg. Am..

[7] Wang T., Ding W. (2020). Risk factors for adjacent segment degeneration after posterior lumbar fusion surgery in treatment for degenerative lumbar disorders: A meta-analysis. J. Orthop. Surg. Res..

[8] Stokes I.A., Iatridis J.C. (2004). Mechanical Conditions That Accelerate Intervertebral Disc Degeneration: Overload *Versus* Immobilization. Spine.

[9] Gruca A. (1956). La patogenie de la scoliosis idiopatique. Rev. De Chir. Ortop..

[10] Gruca A. (1958). The pathogenesis of idiopathic scoliosis. J. Bone Jt. Surg..

[11] Dwyer A.F., Schafer M.F. (1974). Anterior approach to scoliosis. Results of treatment in fifty-one cases. J. Bone Jt. Surg. Br..

[12] Hammerberg K.W., Zielke K. VDS instrumentation for idiopathic thoracic curvatures. Proceedings of the Annual Meeting of the American Academy of Orthopedic Surgeons.

[13] Kaneda K., Shono Y., Satoh S., Abumi K. (1997). Anterior correction of thoracic scoliosis with Kaneda anterior spinal system. A preliminary report. Spine.

[14] Halm H.F., Liljenqvist U., Niemeyer T., Chan D.P.K., Zielke K., Winkelmann W. (1998). Halm-Zielke instrumentation for primary stable anterior scoliosis surgery: Operative technique and 2-year results in ten consecutive adolescent idiopathic scoliosis patients within a prospective clinical trial. Eur. Spine J..

[15] Betz R.R., Harms J., Clements D.H., Lenke L.G., Lowe T.G., Shufflebarger H.L., Jeszenszky D., Beele B. (1999). Comparison of anterior and posterior instrumentation for correction of adolescent thoracic idiopathic scoliosis. Spine.

[16] Ogden J., Ganey T., Sasse J., Neame P., Hibelink D., Weinstein S. (1993). Development and maturation of the axial skeleton. The Pediatric Spine.

[17] Villemure I., Stokes I.A. (2009). Growth Plate Mechanics and Mechanobiology. A Survey of Present Understanding. J. Biomech..

[18] Stokes I.A.F. (2002). Mechanical Effects on Skeletal Growth. J. Musculoskelet. Neuronal Interact..

[19] Ballal M.S., Bruce C.E., Nayagam S. (2010). Correcting genu varum and genu valgum in children by guided growth: Temporary hemiepiphysiodesis using tension band plates. J. Bone Jt. Surg. Br..

[20] Chay E., Patel A., Ungar B., Leung A., Moal B., Lafage V., Farcy J.P., Schwab F. (2012). Impact of Unilateral Corrective Tethering on the Histology of the Growth Plate in an Established Porcine Model for Thoracic Scoliosis. Spine.

[21] Bylski-Austrow D.I., Wall E.J., Glos D.L., Ballard E.T., Montgomery A., Crawford A.H. (2009). Spinal hemiepiphysiodesis decreases the size of vertebral growth plate hypertrophic zone and cells. J. Bone Jt. Surg..

[22] Newton P.O., Glaser D.A., Doan J.D., Farnsworth C.L. (2013). 3D Visualization of Vertebral Growth Plates and Disc: The Effects of Growth Modulation. Spine Deform..

[23] Marks D.S., Iqbal M.J., Thompson A.G., Piggott H. (1996). Convex spinal epiphysiodesis in the management of progressive infantile idiopathic scoliosis. Spine.

[24] Nilsonne U. (1969). Vertebral epiphysiodesis of the thoracic curve in the treatment of idiopathic scoliosis. Acta Orthop. Scandinav..

[25] Betz R.R., Ranade A., Samdani A.F., Chafetz R., D’Andrea L.P., Gaughan J.P., Asghar J., Grewal H., Mulcahey M.J. (2010). Vertebral body stapling: A fusionless treatment option for a growing child with moderate idiopathic scoliosis. Spine.

[26] Newton P.O. (2020). Spinal growth tethering: Indications and limits. Ann. Transl. Med..

[27] Alanay A., Yucekul A., Abul K., Ergene G., Senay S., Ay B., Cebeci B.O., Dikmen P.Y., Zulemyan T., Yavuz Y. (2020). Thoracoscopic Vertebral Body Tethering for Adolescent Idiopathic Scoliosis. Follow-up Curve Behavior According to Sanders Skeletal Maturity Staging. Spine.

[28] Roaf R. (1960). Vertebral growth and its mechanical control. J. Bone Jt. Surg. Br..

[29] Newton O.P., Upasani V.V., Farnsworth C.L., Oka R., Chambers R.C., Dwek J., Kim J.R., Perry A., Mahar A.T. (2008). Spinal growth modulation with use of a tether in an immature porcine model. J. Bone Jt. Surg. Am..

[30] Roaf R. (1963). The Treatment of Progressive Scoliosis by Unilateral Growth-Arrest. J. Bone Jt. Surg. Br..

[31] Denis F. (1983). The three column spine and its significance in the classification of acute thoracolumbar spinal injuries. Spine.

[32] Miyanji F., Pawelek J., Nasto L.A., Rushton P., Simmonds A., Parent S. (2020). Safety and efficacy of anterior vertebral body tethering in the treatment of idiopathic scoliosis. Bone Jt. J..

[33] Senkoylu A., Cetinkaya M. (2017). Correction manoeuvres in the surgical treatment of spinal deformities. EFORT Open Rev..

[34] Newton O.P., Kluck D.G., Saito W., Yaszay B., Bartley C.E., Bastrom T.P. (2018). Anterior Spinal Growth Tethering for Skeletally Immature Patients with Scoliosis: A Retrospective Look Two to Four Years Postoperatively. J. Bone Jt. Surg. Am..

[35] Takahashi Y., Saito W., Yaszay B., Bartley C.E., Bastrom T.P., Newton P.O. (2021). Rate of Scoliosis Correction After Anterior Spinal Growth Tethering for Idiopathic Scoliosis. J. Bone Jt. Surg. Am..

[36] Krakow R.A., Magee L.C., Cahill P.J., Flynn J.M. (2021). Could have tethered: Predicting the proportion of scoliosis patients most appropriate for thoracic anterior spinal tethering. Spine Deform..

[37] Sanders J.O., Khoury J.G., Kishan S., Browne R.H., Mooney J.F., Arnold K.D., McConnell S.J., Bauman J.A., Finegold D.N. (2008). Predicting scoliosis progression from skeletal maturity: A simplified classification during adolescence. J. Bone Jt. Surg. Am..

[38] Crawford H.C., Lenke L.G. (2010). Growth modulation by means of anterior tethering resulting in progressive correction of juvenile idiopathic scoliosis: A case report. J. Bone Jt. Surg. Am..

[39] Samdani F.A., Ames R.J., Kimball J.S., Pahys J.M., Grewal H., Pelletier G.J., Betz R.R. (2014). Anterior vertebral body tethering for idiopathic scoliosis: Two-year results. Spine.

[40] Tsirikos A.I., Loughenbury P.R. (2018). Single rod instrumentation in patients with scoliosis and co-morbidities: Indications and outcomes. World J. Orthop..

[41] Stagnara P., DE Mauroy J.C., Dran G., Gonon G.P., Costanzo G., Dimnet J., Pasquet A. (1982). Reciprocal angulation of vertebral bodies in a sagittal plane: Approach to references for the evaluation of kyphosis and lordosis. Spine.

